# The immediate impacts of TV programs on preschoolers' executive functions and attention: a systematic review

**DOI:** 10.1186/s40359-024-01738-1

**Published:** 2024-04-24

**Authors:** Sara Arian Namazi, Saeid Sadeghi

**Affiliations:** Institute for Cognitive and Brain Sciences, Shahid Beheshti University, Tehran, Iran

**Keywords:** Attention, Television, Preschool, Fast-paced TV program, Slow-paced TV program, Fantasy, Executive function, Systematic review

## Abstract

**Background:**

Previous research has presented varying perspectives on the potential effect of screen media use among preschoolers. In this study, we systematically reviewed experimental studies that investigated how pacing and fantasy features of TV programs affect children's attention and executive functions (EFs).

**Methods:**

A systematic search was conducted across eight online databases to identify pertinent studies published until August 2023. We followed the PRISMA (Preferred Reporting Items for Systematic Reviews and Meta-Analysis) guidelines.

**Results:**

Fifteen papers involving 1855 participants aged 2–7 years fulfilled all the inclusion criteria for this review and were entered into the narrative synthesis. Despite the challenge of reaching general conclusions and encountering conflicting outcomes, a nuanced analysis reveals distinct patterns within various subgroups. The impact of pacing on attention is discernible, particularly in bottom-up attention processes, although the nature of this effect remains contradictory. Conversely, consistent findings emerge regarding top-down attention, suggesting any impact. Moreover, a subgroup analysis of different EF components yields valuable insights, highlighting the negative effect of fantasy on inhibitory control within the EF framework.

**Conclusion:**

The complexity of these outcomes highlights the need for further research, considering factors such as content, child-specific characteristics, environmental factors, and methodological approaches. These findings collectively emphasize the necessity of conducting more comprehensive and detailed research, especially in terms of the underlying mechanisms and their impact on brain function.

## Introduction

In the last few decades, the advancement of technology has made digital devices a significant part of children's lives [1]. Children are now using digital devices at a younger age as devices are more readily available at home, school, and in society as a whole [2–4]. Studies have shown that excessive screen time is associated with obesity and sleep problems, as well as lowered social and motor development scores in young children [5, 6]. In recent years, researchers have been studying the interaction between digital devices and children's cognitive development [7].

The term “digital devices” refers to devices that can create, generate, share, communicate, receive, store, display, or process information, including, but not limited to, laptops, tablets, desktops, televisions (TVs), mobile phones, and smartphones [8]. TV is one of the digital devices well-studied for its effects on children and refers to shows (e.g. live-action, puppets, …) and cartoons that children watch on TVs and other touchscreen devices [9]. The effects of TV content are determined by many factors, including fantastical content and the program's pacing [10]. Pacing refers to how fast audio and visual elements change [11]. Video pace can be assessed through varying filming techniques, like changing the camera's perspective [12] or transitioning between scenes [13]. The concept of fantasy is about phenomena that defy the laws of reality, such as Superman [14].

Recent studies have examined whether TV (the pace and fantasy events in the programs) affects children's cognitive development, particularly regarding attention and executive functions (EFs). Attention is a multifaceted cognitive mechanism characterized by the allocation of resources towards distinct stimuli or tasks, thereby facilitating heightened processing and perception of relevant information [15, 16]. There is a difference between attention and higher cognitive functions (e.g., executive functions). The attention process occurs between perception, memory, and higher cognitive functions. In this way, information can flow from perception to memory and higher cognitive functions and vice versa [17, 18]. Many models have been developed to explain attention ability, and some of these models include components that are related to EF. EFs encompass a spectrum of cognitive processes essential for solving goal-oriented problems. This term comprises diverse higher-order cognitive functions including reasoning, working memory, problem-solving, planning, inhibitory control, attention, multitasking, and flexibility [19–21]. These functions are often referred to as "cool" EF, as the underlying cognitive mechanisms operate with limited emotional arousal [22]. In contrast, "hot" EF involves emotion or motivation, such as rewards or punishment tacking [22, 23]. Within this classification, two subsets encompass basic EFs like working memory, inhibition, attention control, and cognitive flexibility, along with higher-order (higher-level) EFs such as reasoning, problem-solving, and planning, which stem from these basic ones [20].

Due to the complexity of the topic, studies investigating the relationship between TV programs and attention or EF have adopted diverse assessment methods. In some studies, children's involvement in tasks during free play or direct testing has been used to measure attention [24]. Another substantial portion of these studies adopted the model of EF proposed by Miyake et al. [25], which divided EF into three components: inhibitory control (the ability of a person to inhibit dominant or automatic responses in favor of less prominent data), working memory (the capacity to hold and manipulate various sets of information) and flexibility (shifting attention) [10, 26, 27]. Alternatively, some studies have measured EF through two dimensions: "hot" and "cool" [13, 14]. Another subset of related research has focused on higher-order EF tests, encompassing domains such as planning and problem-solving. Additionally, a few studies have measured EF in a very general way, with tasks that address different parts of EF (assessed through tasks involving color separation or completing puzzles as quickly as possible) [28].

As an illustration, Cooper et al. [12] investigated the influence of pacing on attention using a direct task and demonstrated a positive effect on performance in EF tasks. In another study by Lillard and Peterson [13], the impact of pacing on Cool EF was investigated, revealing a reduced performance in EF tasks after exposure to fast-paced programs. Regarding higher-order EFs, the 2022 study [29] concluded that exposure to a fast-paced TV program did not immediately affect children's problem-solving abilities. Moreover, Jiang et al. [26] evaluated EFs based on Miyake's model, indicating that fantastical events negatively affected inhibitory control and flexibility, whereas working memory remained unaffected.

A limited capacity model and the attention system are essential for explaining the underlying mechanisms behind how TV pacing impacts children's cognitive performance. It has been proposed that fast-paced programs, which are characterized by rapid changes in the scene, capture attention in a bottom-up manner through orienting responses to scene changes, primarily engaging sensory rather than the prefrontal cortex [30, 31]. In this way, fast-paced programs could overwhelm cognitive resources, aligning with the "overstimulation hypothesis" [32–34]. This hypothesis posits that exposure to such programs may lead the mind to anticipate high levels of stimulation, which can reduce children's attention spans and influence their performance [31, 32]. Furthermore, a study by Carey [35] revealed young children's anticipations about the occurrence of events. Likewise, Kahneman [36] proposed the concept of a single pool of attentional resources and suggested that processing fantastical events overloads limited cognitive resources. Watching TV programs engages the bottom-up cognitive processing system. Consequently, the top-down cognitive processing system may be delayed in re-engaging in subsequent cognitive tasks after program viewing [14]. This suggests that exposure to fast-paced and fantastical TV programs has temporary effects on children's attention and executive functioning.

Research examining the immediate impact of these two features on children's attention and EF has yielded conflicting outcomes. Several studies indicate that fast-paced television programs have a negative effect on children's attention and EFs [13, 28, 37, 38]. In contrast, some studies have shown positive results [12, 39], while other studies found no significant impact [14, 27, 29, 40]. Similar findings are observed for the fantasy feature. Some studies have shown that higher levels of fantastical content led to lower performance on cognitive tests [10, 14, 26, 27, 41, 42], while contrary findings are also reported [39, 43].

Therefore, it remains unclear how television content affects children's attention and EFs. Due to this, it is necessary to identify any gaps in the prior research, which can lead to effective strategies to investigate TV programs' effects. Previous reviews: (1) summarized the relationship between screen time and EF [44]; (2) adopted a comprehensive approach by combining diverse research methodologies, yet omitted some recent studies [24]; and (3) summarized the influence of media on self-regulation, although they emphasized several studies, overlooking a subset of investigations concerning the immediate impact of TV programs [45]. None of these reviews have specifically focused on the outcomes of experimental research. To investigate the effects of programs, experimental studies seem to be a more accurate research method. Experiments allow the control of certain variables and manipulation of an independent variable (such as the pace of the program and fantasy). This review aims to explore the immediate impact of TV pacing and fantasy features on children's attention and EF, as well as the potential factors contributing to the variations in outcomes.

## Methods

### Search strategy

This systematic review follows the guidelines set by the Preferred Reporting Items for Systematic Reviews and Meta-analyses (PRISMA) protocol [46]. We searched eight online databases on 2 August 2023: APA PsycARTICLES, Cochrane Library, EBSCO (APA PsycINFO), Google Scholar (limited to first three pages), Ovid, ProQuest, PubMed (MedLINE), and Web of Science. The search strategy utilized the article abstract and ignored the date and language restrictions: child* OR preschool* AND television OR TV OR cartoon AND executive function OR attention OR inhibit* OR flexibility OR working memory AND immediate* OR short-term OR pace OR fantasy. This strategy was tailored to suit the requirements of each database. Additionally, to account for any potentially overlooked studies, citation searching was conducted for the Lillard et al. [14] article on Google Scholar on 7 August 2023. However, only studies with relevant titles and abstracts were included in the review screening.

### Study selection

**Fig. 1:**
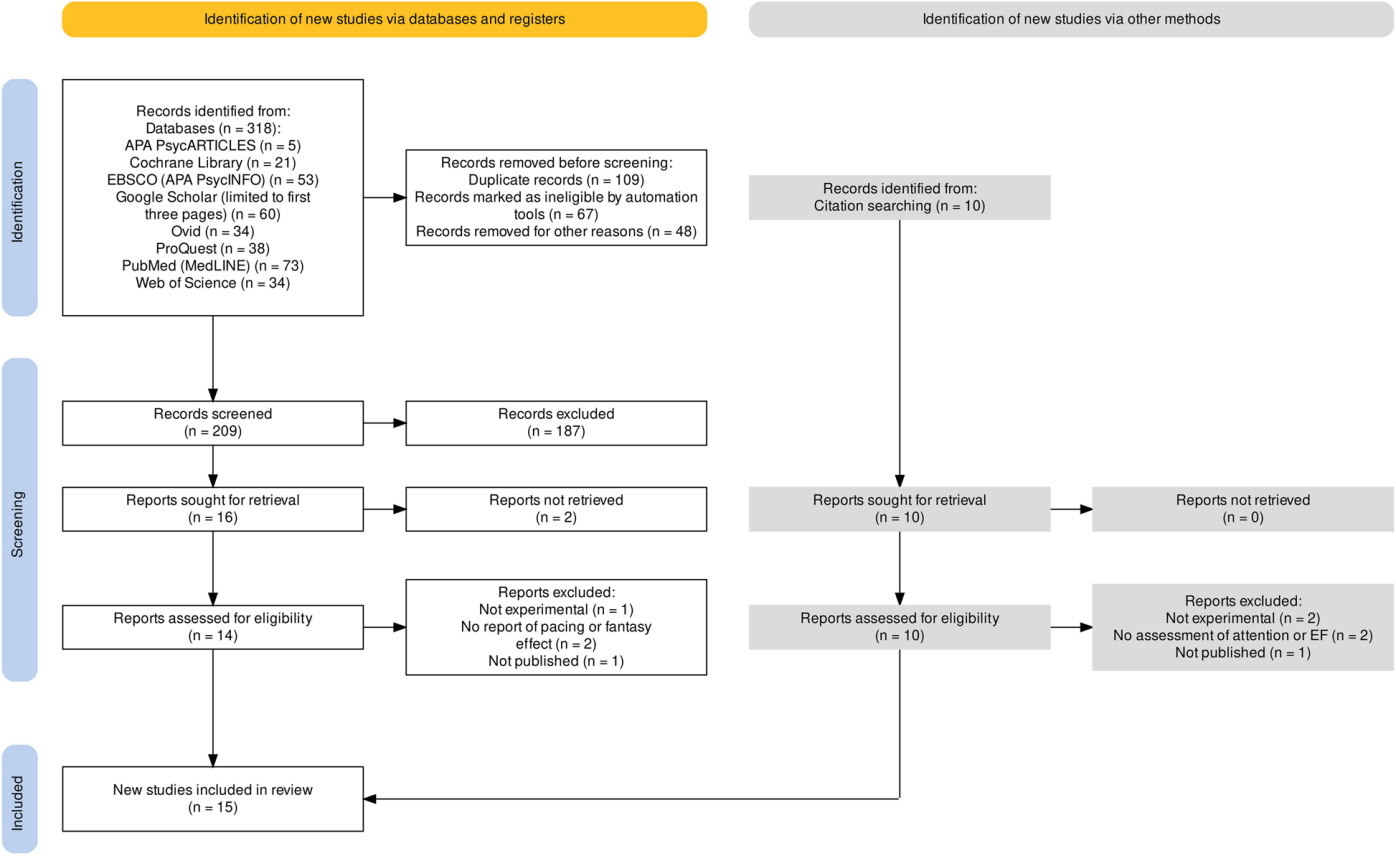
PRISMA flow diagram [48] showing the number of studies that were removed at each stage of the literature search

The studies had to meet these criteria to be included in the review: (1) participants were children younger than seven years (preschool); (2) the study assessed the impact of TV programs on children's attention or EFs; (3) the independent variable was the exposure to a TV program (including cartoons and non-animated programs, while excluding advertisements), with immediate measurement of its impact on children's attention or EF; (4) the study measured the effect of pacing and fantasy features present in TV programs; (5) the study had an experimental design; and (6) the research was published as journal articles in English. Furthermore, any study where a participant had been diagnosed with a disorder was excluded from the review. The initial identification yielded 328 potentially relevant studies, from which 67 duplicates were eliminated using EndNote 20's automated tool [47]. Additionally, the manual review led to the elimination of 42 more duplicates, while six non-English studies were further removed. The remaining 203 studies were screened for title and abstract relevance. Subsequently, two screeners reviewed the full text and included 15 as eligible studies. Any conflict between screeners regarding eligibility was resolved through discussions. The PRISMA chart that summarizes these processes can be seen in Fig. 1.

### Data extraction and synthesis

The relevant data from the selected studies were extracted on a form by two reviewers, and any conflict was resolved through discussion. The data extraction form had information about the characteristics of each study: authors’ names, titles of manuscripts, publication dates, sample sizes, the mean and standard deviation of participant ages, the proportion of females within the sample, TV program name, features and length, type of cognitive functions (EFs or attention) measured in the study along with their assessment methods and variables used for controlling or checking differences between groups. Additionally, eligible outcomes were as follows: the effect of fast and slow-paced TV programs, the effect of fantastical and realistic TV programs, and variable interactions. In our research, the data synthesis was conducted using narrative synthesis for the included studies. This choice was driven by the conflicting results observed across the various studies. Although a single reviewer composed the narratives, all decisions were reached through discussions involving two reviewers.

### Quality assessment

The evaluation of study quality was conducted utilizing the Downs and Black [49] checklist, which has 27 items. However, not all of these items apply to every type of study design. Following a similar approach as Uzundağ et al. [45], for the experimental studies, a subset of 21 relevant items was employed. The study's quality check result can be found in Table 1.

**Table 1:** Concise overview of TV programs' effects on children's attention studies

Study	Participants	TV program’s		Conditions	Attention assessment		Conclusion	Quality
		Feature	Length		Pre-viewing	Post-viewing		
Anderson et al. [40]	*n* = 72 4-year-olds	Pace	40 min	1. Sesame Street (fast-paced; edited version) 2. Sesame Street (slow-paced; edited version) 3. Read a storybook by a parent (control)	––-	• Replacement Puzzle Test • 10- minute Free-play observations	No immediate effect of pacing	17/21
Geist and Gibson [37]	*n* = 62 4- to 6-year-olds	Pace	30 min	1. Mighty Morphin’ Power Rangers (fast-paced) 2. Mister Rogers’ Neighborhood (slow-paced) 3. Free-play (control)	––-	• Free-play: Number of task changes observations • Free-play: Time on-task observations	Negative effect of fast-paced TV programs	15/21
Cooper et al. [12]	*n* = 37 4- to 7-year-olds	Pace	3.5 min	1. Narration of Winnie at the Seaside book (fast-paced; edited version) 2. Narration of Winnie at the Seaside book (slow-paced; edited version)	––-	• Attention Networks Task	Positive effect of fast-paced TV programs Age x pacing interaction has a significant effect	13/21
Kostyrka-Allchorne et al. [38]	*n* = 70 2- to 4.5-year-olds	Pace	4 min	1. Narration of Winnie at the Seaside book (fast-paced; edited version) 2. Narration of Winnie at the Seaside book (slow-paced; edited version)	Free-play: number of toy changes observations	• Free-play: number of toy changes observations	Negative effect of fast-paced TV programs on attention	16/21
Kostyrka-Allchorne et al. [39]	*n* = 187 3.5- to 5-year-olds	Pace and Fantasy	5–6 min	1. Narration of Room on the Broom book (fast and fantastical; edited version) 2. Narration of Room on the Broom book (slow and fantastical; edited version) 3. narration of Charlie and Lola book (fast and non-fantastical; edited version) 4. narration of Charlie and Lola book (slow and non-fantastical; edited version)	––-	• Continuous performance test (CPT)	No immediate effect of pace or fantasy on attention Fantasy x pacing interaction has a significant effect on attention: positive effect of fast-paced	18/21

## Results

### Overview

A total of 1855 children aged between two and seven years participated in the 15 studies (49.43%, female). Among these studies, seven exclusively investigated the impact of pacing, with four exploring its effects on attention and three on EF. Additionally, three studies examined the impact of pacing and fantasy, with only one focusing on attention, while five studies specifically concentrated on the fantasy effect on EF. The sample sizes varied from 20 to 279 participants, while the duration of video exposure ranged from 3.5 to 40 min. The mean age of participants, as reported in 13 studies, was 59.56 months (SD = 9.94). Notably, only seven studies involved a pre-test, eight studies controlled for the overall media exposure, and four considered socioeconomic status (SES).

Five of the conducted studies measured attention. As for EF, the studies explored a diverse range of EF components: inhibitory control was measured in five studies, cognitive flexibility in four, working memory in three, composite cool EF in three, and hot EF in two, with one study each dedicated to measuring planning, problem-solving, and general EF (motor EF). For assessment, attention was operationalized through either the observation of children's behavior during free play or direct task measurement. In all these studies, EF was directly assessed through various tasks.

### Pace

#### Attention

Experimental investigations into the impact of TV program pacing on preschoolers' attention have yielded inconsistent outcomes. Among the initial two studies, fast-paced TV programs negatively impacted children’s attention. Geist and Gibson [37] examined the effects of rapid TV program pacing on 62 children aged 4 to 6. Their findings demonstrated that children exposed to a fast-paced program displayed more frequent activity switches and allocated less time to tasks during the post-viewing period, in contrast to the control group. This pattern was interpreted as indicative of a shortened attention span in children. However, it cannot be definitively determined whether the observed negative impact could be attributed to content, pacing, or an interplay of both factors. Furthermore, no pre-viewing attention test was included, which complicates the interpretation of the results. To address the pacing/content dilemma, Kostyrka-Allchorne et al. [38] adopted the methodology employed by Cooper et al. [12]. They created experimental videos with identical content, varying only in the number of edits (pace). In this study, 70 children aged 2 to 4.5 years were exposed to one of two 4-min edited videos featuring a narrator reading a children's story. The fast-paced group displayed more frequent shifts of attention between toys than the slow-paced group, despite the lack of initial behavioral differences between the groups before watching the videos. By coping with the pacing/content issue and incorporating younger participants, this study provides insights, albeit with video durations that notably differ from typical children's program episodes.

In contrast to the studies mentioned earlier, the subsequent two studies propose that fast-paced TV programs may not significantly impact or might even yield positive ones on children's attention. To elaborate, Anderson et al. [40] initiated their research by subjecting 4-year-old children to a 40-min fast-paced or slow-paced version of *Sesame Street*, while a control group listened to a parent reading a story. The findings failed to provide substantial support for the immediate effects of TV program pacing on the behavior and attention of preschoolers. In a subsequent study, Cooper et al. [12] presented a 3.5-min video of a narrator reading a story to children aged 4 to 7. This investigation employed edited versions of the video to create both fast-paced and slow-paced versions with identical content. Through applying an attention networks task, post-viewing evaluation alerting, orienting, and executive control. The outcomes revealed that even a very brief exposure to programs can impact children's orienting networks and error rates. Moreover, a noteworthy interaction emerged between age and pacing: 4-year-olds displayed lower orientation scores in the fast-paced group compared to the slow-paced one, while the reverse occurred for the 6-year-olds. In summary, these two studies maintained consistent video content by manipulating pacing, focusing solely on evaluating the pacing effect. However, it's important to acknowledge that Anderson et al. [40] utilized TV programs with a slower pace than contemporary ones, and Cooper et al. [12] subjected children to programs for 3.5 min—considerably shorter than the typical time children spend watching TV programs [14]. Refer to Table 1 for a concise overview of attention studies.

#### EF

Regarding EF, research examining the influence of pacing has also produced inconsistent outcomes. Lillard and Peterson [13] explored the immediate impact of fast-paced TV content on the EF of 60 four-year-olds. In this study, participants were exposed to a 9-min cartoon episode (fast or slow-paced content) or were engaged in drawing (serving as the control condition). The results indicated that children who viewed the fast-paced cartoon performed notably poorer on a post-viewing Cool and Hot EF assessment when compared to the other groups. This finding underscores the significant influence of pacing on children's EF. Additionally, Sanketh et al. [28] investigated the impact of a TV program's pacing on children's motor EF. Involving a sample of 279 four- to six-year-olds, the study began with a pre-viewing test to ensure developmental equivalence among participants. The findings revealed that children exposed to the fast-paced cartoon exhibited slower performance on motor EF tasks compared to their counterparts in the other two groups. This outcome suggested that ten minutes of viewing a fast-paced cartoon yielded an immediate negative impact on the motor EF of 4- to 6-year-old children. However, it's important to note that these two studies could not differentiate between the effects of pacing and content.

In contrast to these studies, Rose et al. [29] more recently delved into the effects of TV program pacing on problem-solving abilities through ecologically valid research. In this study, each child underwent exposure to both fast and slow programs during two distinct sessions to ensure comparability and control over other variables. Notably, no significant differences emerged in the problem-solving task between the fast and slow programs. The study identified no significant differences in problem-solving performance between the two pacing conditions. However, following exposure to the fast-paced program, both age groups demonstrated a non-significant increase in EF scores (p = 0.71). Additionally, the study by Rose et al. [29] aimed to ensure content parity between the fast and slow programs, leading to a smaller pacing difference compared to certain other studies. Refer to Table 2 for a concise overview of EF studies.

**Table 2:** Concise overview of TV programs' effects on children's EF studies

Study	Participants	TV program’s		EF type	Conditions	EF assessment		Conclusion	Quality
		Feature	Length			Pre-viewing	Post-viewing		
Lillard and Peterson [13]	*n* = 60 4-year-olds	Pace	9 min	Cool EF Hot EF	1. SpongeBob SquarePants (fast-paced) 2. Caillou (slow-paced) 3. drawing (control)	Parent Questionnaires	Cool EF: • Tower of Hanoi task • Head, Toes, Knees, and Shoulders task (HTKS) • Backward Digit Span Hot EF: • Delay-of-Gratification (DoG)	Negative effect of fast-paced TV programs on Cool EF and Hot EF	19/21
Lillard et al. [14]	Study 1: *n* = 160 4- and 6-year-olds	Pace and Fantasy combined	11 min	Cool EF Hot EF	1. SpongeBob SquarePants (fast-paced & fantastical) 2. Fan Boy and Chum Chum (fast-paced & fantastical) 3. Arthur (slow-paced & non-fantastical) 4. Free-play with toys (control)	Parent Questionnaires	Cool EF: • Tower of Hanoi task • Head, Toes, Knees, and Shoulders task (HTKS) • Auditory Working Memory Creativity: • Functional Fixedness task Hot EF: • Delay-of-Gratification (DoG)	Negative effect of fast-paced and fantastical TV programs on Cool EF No effect of fast-paced and fantastical TV programs on Hot EF, but slow-paced and non-fantastical ones have a positive effect No immediate effect of pace and fantasy on creativity	17/21
	Study 2: *n* = 60 4-year-olds	Pace and Fantasy combined	22 min	Cool EF	1. SpongeBob SquarePants (fast-paced & fantastical) 2. Martha Speaks video (fast-paced & fantastical) 3. Martha Speaks Book (control)	Parent Questionnaires	• Tower of Hanoi task • Dimensional Changes Card Sort (DCCS) • Auditory Working Memory • Luria’s Hand game	Negative effect of fast-paced and fantastical TV programs on EF	17/21
	Study 3: *n* = 80 4-year-olds	Pace and Fantasy	8–9 min	Cool EF	1. SpongeBob SquarePants (fast-paced & fantastical) 2. Phineas and Ferb (fast-paced & non-fantastical = 0.13) 3. Little Einsteins (slow-paced & fantastical) 4. Little Bill (slow-paced & non-fantastical)	Parent Questionnaires • Dimensional Changes Card Sort (DCCS) • Auditory Working Memory • Luria’s Hand game • Gift Wrap DoG	Cool EF: • Tower of Hanoi task • Head, Toes, Knees, and Shoulders task (HTKS) • Auditory Working Memory • Day/Night task Hot EF: • Forbidden Toy DoG	No immediate effect of pacing Negative effect of fantastical TV programs on Cool EF * The two Hot EF tasks were left out of all analyses	17/21
Sanketh et al. [28]	*n* = 279 4- to 6-year-olds	Pace	10 min	Motor EF	1. Tom and Jerry (fast-paced) 2. Barney cartoon (slow-paced) 3. Painting with crayons	• Seguin Form Board	• Namely Color Match • Two-piece Puzzle • Separating Colored Beads	Negative effect of fast-paced TV programs on motor EF	16/21
Li et al. [42]	Study 1: *n* = 72 4- and 6-year-olds	Fantasy	11 min	Inhibitory control	1. Dr. Panda in Space videoclip (fantastical) 2. Dr. Panda in Space game (fantastical)	• Go-No-Go task	• Go-No-Go task	Negative effect of fantastical TV programs on inhibitory control, but no effect of fantastical games	13/21
	Study 3: *n* = 72 4- and 6-year-olds	Fantasy	11 min	Inhibitory control	1. Dr. Panda in Home videoclip (non-fantastical) 2. Dr. Panda in Home game (non-fantastical)	• Go-No-Go task	• Go-No-Go task	Positive effect of non-fantastical TV programs and games on inhibitory control	14/21
Jiang et al. [26]	*n* = 143 5-year-olds	Fantasy	12 min	Inhibitory control Working memory Flexibility	1. Pleasant Goat and Big Big Wolf ep 10 (high-fantasy) 2. Pleasant Goat and Big Big Wolf ep 57 (mid-fantasy) 3. Pleasant Goat and Big Big Wolf ep 20 (low-fantasy)	Parent Questionnaires Peabody Picture Vocabulary test	Inhibitory control: • NIH Toolbox FICA test Working memory: • NIH Toolbox LSWM Flexibility: • NIH Toolbox DCCS	Negative effect of mid-fantasy TV programs on inhibitory control and cognitive flexibility No immediate effect of fantasy on working memory	18/21
Kostyrka-Allchorne et al. [39]	*n* = 187 3.5- to 5-year-olds	Pace and Fantasy	5–6 min	Inhibitory control	1. Narration of Room on the Broom book (fast and fantastical; edited version) 2. Narration of Room on the Broom book (slow and fantastical; edited version) 3. narration of Charlie and Lola book (fast and non-fantastical; edited version) 4. narration of Charlie and Lola book (slow and non-fantastical; edited version)	• Day/night task	• Day-night task	Positive effect of fantastical TV programs on inhibitory control, but no effect of pacing	18/21
Rhodes et al. [10]	*n* = 80 5- to 6-year-olds	Fantasy	23 min	Inhibitory control Working memory Flexibility Planning	1. Little Einsteins (fantastical) 2. Little Bill (non-fantastical)	Parent Questionnaires Inhibitory control: • Day/night task Working memory: • Backward Digit Span Flexibility: • Standard Dimensional Change Card Sort Planning: • Tower of Hanoi	Inhibitory control: • Day/night task Working memory: • Backward Digit Span Flexibility: • Standard Dimensional Change Card Sort Planning: • Tower of Hanoi	Negative effect of fantastical TV programs on inhibitory control, cognitive flexibility, and working memory No immediate effect of fantasy on planning	17/21
Li et al. [41]	Study 1: *n* = 90 4- to 6-year-olds	Fantasy	18–19 min	Cool EF	1. Mickey Mouse Clubhouse (non-fantastical) 2. Tom and Jerry (fantastical) 3. Usual classroom activities	Parent Questionnaires	• Day/night task • Backward Digit Span • Flexible item section	Negative effect of fantastical TV programs on EF	16/21
	Study 2: *n* = 20 4- to 6-year-olds	Fantasy	18–19 min	Cool EF	1. Mickey Mouse Clubhouse (non-fantastical) 2. Tom and Jerry (fantastical)	Parent Questionnaires	• Day/night task • Backward Digit Span • Flexible item section	Negative effect of fantastical TV programs on EF Eye tracker: more but shorter eye fixations in the fantastical condition	16/21
	Study 3: *n* = 20 4- to 6-year-olds	Fantasy	18–19 min	Cool EF	1. Mickey Mouse Clubhouse (non-fantastical) 2. Tom and Jerry (fantastical)	Parent Questionnaires	• Day/night task • Backward Digit Span • Flexible item section	Negative effect of fantastical TV programs on EF fNIRS: higher Coxy-Hb in PFC in fantastical condition	16/21
Fan et al. [27]	*n* = 218 4- to 7-year-olds	Pace and Fantasy	11 min	Inhibitory control Working memory Flexibility	1. SpongeBob (fast-paced and fantastical) 2. Tom and Jerry (slow-paced and fantastical) 3. Boonie Bear (fast-paced and non-fantastical) 4. Big Head Son and Little Head Father (slow-paced and non-fantastical)	Parent Questionnaires	Inhibitory control: • Day/night Stroop task Working memory: • Backward Digit Span Flexibility: • Flexible item section	No immediate effect of pacing Negative effect of fantastical TV programs on inhibitory control, cognitive flexibility, and working memory Age x fantasy interaction has a significant effect on inhibitory control	19/21
Rose et al. [29]	*n* = 41 3- and 4-year-olds	Pace	15 min	Problem-solving	Postman Pat: 1. Postman Pat ep Postman Pat and the Robot (slow-paced) 2. Postman Pat ep Flying Christmas Stocking (fast-paced)	Parent Questionnaires	• Block Buddies	No immediate effect of pacing on problem-solving	18/21
Wang and Moriguchi [43]	*n* = 32 3- to 6.5-year-olds	Fantasy	5 min	Flexibility	1. Dr. Panda in Space videoclip (fantastical) 2. Dr. Panda in Space game (fantastical)	• Standard Dimensional Change Card Sort	• Standard Dimensional Change Card Sort	No immediate effect of fantasy on flexibility fNIRS: No immediate effect of fantasy on the brain	15/21

#### Fantasy

Continuing the exploration of the distinct impacts of TV program content, particularly in the context of fantasy, Lillard et al. [14] introduced a novel dimension to the discussion. The concept of "fantastical" versus "non-fantastical" (also termed "realistic" or "unrealistic") content emerged as a notable category within TV programming. This idea prompted three separate research studies, all aiming to disentangle the effects of pacing from fantasy on children's EF. To address this inquiry, all three studies employed a common approach, utilizing four TV programs that varied along two dimensions: fast and fantastical, fast and non-fantastical, slow and fantastical, or slow and non-fantastical. Of these three, only one study focused on attention.

#### Attention

Kostyrka-Allchorne et al. [39] conducted a study in 2019 with 187 children aged 3.5 to 5 years, exposing them to 5-min self-produced videos. Their findings indicated that there is a significant interaction between pacing and fantasy, while neither factor displayed an individual effect. Notably, exposure to the fast-paced video led to quicker responses, but only when the story was non-fantastical. However, due to the brief length of the videos, it's uncertain if the stimuli adequately challenged cognitive resources (see Table 1).

#### EF

There is a more extensive body of literature on EF (all three mentioned studies) that accurately separates the effect of pace from fantasy. The outcomes of these studies indicated a lack of influence from pacing, while the impact of fantasy and the interplay between pacing and fantasy yielded conflicting results. Lillard et al. [14] conducted three distinct studies to test their hypotheses, building upon their prior research findings. Study 1 involved diverse videos with an extended duration (11 min) compared to the 2011 study [13], focusing on 4- and 6-year-olds. The findings indicated that children's Cool EF scores were notably lower in the two fast and fantastical conditions compared to the control group. Conversely, children in the slow and non-fantastical condition performed better in the hot EF task. Study 2 aimed to discern whether solely fast and fantastical entertainment TV programs, as opposed to educational ones, influenced children's EF. The results indicated that even when designed with educational intent, watching a fast and fantastical TV program led to lower EF scores than reading a book. Additionally, the EF performance following exposure to the educational program was similar to that of the entertaining program. In the final study, Lillard et al. [14] aimed to differentiate the contributions of fantasy versus pacing (fast or slow). The analysis revealed that fantastical content has an impact on EF, although fast-paced did not show a similar effect. However, this particular study focused on a single age group without considering potential age-related nuances in the development of EF.

Moreover, Kostyrka-Allchorne et al.'s [39] findings indicated that children in two fantastical conditions had higher inhibitory control scores than those in the alternative condition, yet no discernible pacing effect was observed. In a parallel vein, within the same investigative framework as Lillard et al.’s [14] Study 3, Fan et al. [27] explored the age-related influence on the impact of TV program features on EF of children aged 4 to 7 years. Employing four 11-min cartoons for exposure, the study revealed that following fantastical TV program viewing, children's performance on subsequent EF tasks declined. Albeit, the pacing did not exert a comparable effect. The most significant interaction emerged between fantasy and age, indicating a heightened impact of fantasy on inhibitory control among younger children. Unlike the earlier studies, this study emphasized EF development and encompassed a broader age range of children. In summation, these three research studies reveal inconsistent results. To address the novelty aspect inherent in EF tests, Fan et al. [27] adopted parent questionnaires to account for pre-viewing EF levels. In contrast, the other two studies incorporated at least one task during the pre-viewing session to assess EF.

Expanding upon the findings of Lillard et al. [14], subsequent studies focused exclusively on the impact of fantasy, omitting the pacing feature. Out of the five studies, four of them collectively suggest that fantastical TV programs tend to exert a negative impact on children's EF.

Li et al. [42] undertook a comparative study to assess the effects of viewing versus interacting with fantastical or non-fantastical events on inhibitory control. Through two experimental studies, participants were involved in a video game or a video clip showcasing identical events from the game. The findings indicated that watching fantastical programs led to a reduction in inhibitory control, while interaction with them did not produce a similar effect. Moreover, children in the game condition perceived the fantastical events to be less fantastical. Notably, inhibitory control showed improvement after both watching and interacting with non-fantastical content. It is worth noting that while this study employed direct tasks to address pre-viewing EF levels, the number of fantastical events was not standardized and varied across programs and game conditions. To refine the understanding of the fantasy effect, Jiang et al. [26] introduced three levels of fantasy in their investigation. The findings revealed that working memory scores did not significantly differ across conditions. However, a nonlinear pattern emerged about the effects of fantasy on inhibitory control and cognitive flexibility, with children in the mid-fantasy group demonstrating comparatively poorer performance. Notably, the potential moderating influence of gender on the relationship between fantastical events and EF lacked conclusive evidence. Continuing from the groundwork laid by Lillard et al. [14], Rhodes et al. [10] undertook a study investigating the impact of fantasy on 80 children aged 5 to 6 years. Employing two complete episodes of cartoons utilized by Lillard et al. [14], they revealed that children in the fantastical condition exhibited lower performance on inhibition, working memory, and cognitive flexibility tasks during the post-viewing session. Notably, the disparity in planning tasks did not yield a statistically significant difference. It is worth highlighting that despite employing cartoons from a different study, they were not matched in terms of pace and language factors, which might have influenced their effect on EF.

In a study aligned with the ones mentioned earlier, Li et al. [41] examined whether watching TV programs featuring fantastical events had a diminishing impact on the post-viewing EF of 4- to 6-year-olds. They exposed 90 children to *Mickey Mouse Clubhouse* (non-fantastical), *Tom and Jerry* (fantastical), or typical classroom activities (control). The outcomes indicated significantly lower scores on behavioral EF tasks for children in the fantastical condition compared to the other groups. In their pursuit, Li et al. [41] additionally conducted supplementary experiments. The analysis of eye tracking data revealed heightened and briefer eye fixations, while fNIRS data indicated elevated Coxy-Hb levels in the prefrontal cortex (PFC) of the fantastical group, aligning with models of limited cognitive resources. Similar to the preceding study, a notable distinction between the two cartoons existed. *Mickey Mouse Clubhouse* constituted one episode with a single narrative, whereas *Tom and Jerry* comprised three distinct episodes with separate stories (episodic narratives). Moreover, the differentiation between fantastical events and comedic violence within Tom and Jerry remains unclear.

Conversely, a recent investigation by Wang and Moriguchi [43], adopting the methodology established by Li et al. [42], presented divergent outcomes. After exposure to fantastical content, 3 to 6.5-year-old children's cognitive flexibility and prefrontal activation were assessed. There were no observable alterations in performance or neural activity. In summary, the initial four studies, each exclusively focused on assessing the impact of fantasy, consistently suggest a negative effect. However, the most recent one and the investigation conducted by Kostyrka-Allchorne et al. [39] produced contrasting outcomes, with one indicating a positive impact and the other showing no discernible effect. It is essential to note that Wang and Moriguchi's [43] study covers a wide age range between 3 and 6.5 years and does not consider the potential effect of age. Additionally, the brief duration spent on fantasy content raises concerns, as it may not have allowed sufficient time for any potential effect. Despite drawing inspiration from the methodology used in Li et al.'s [42] study, the number of fantasy events in this recent study was not standardized.

As a result, the impact of exposure to fantastical TV programs on children's EF remains unclear, while the influence of pacing can be more certainly dismissed (see Table 2). Additionally, in the field of attention, it is not possible to draw conclusions based on the study results for both features.

## Discussion

We conducted the current systematic review to gain a better understanding of how TV programs' pace and fantasy may impact children's attention and EF by synthesizing results from multiple experimental studies. The synthesis of the reviewed studies and their outcomes has highlighted variations in how pacing and fantasy influence attention and different aspects of EF. The discussion will now delve into the potential explanations for these observed effects.

### Attention

#### Pacing

Numerous studies have investigated the influence of pacing on children's attention. Anderson et al. [40] and Kostyrka-Allchorne et al. [39] found no significant effects on attention, while Geist and Gibson [37] and Kostyrka-Allchorne et al. [38] reported a negative impact. In contrast, Cooper et al. [12] observed positive results. To explain these results, it's crucial to look at the methodologies employed in attention measurement. Anderson et al. [40], Geist and Gibson [37], and Kostyrka-Allchorne et al. [38] used child observation during free play, whereas Anderson et al. [40] used the Matching Familiar Figures task, Cooper et al. [12] the Attention Networks Task, and Kostyrka-Allchorne et al. [39] the Continuous Performance Task (CPT).

Observational studies during free play suggest that exposure to fast-paced programs leads to more frequent toy switching in children. This rapid switching corresponds to accelerated bottom-up attention [39]. However, Anderson et al. [40] measurements during free play did not reveal this phenomenon. Additionally, exposure to fast-paced programs may diminish children's capacity for reflective processing [50]. Nevertheless, this effect did not manifest in the results of the Matching Familiar Figures task. Anderson et al. [40] showed that neither reflection nor impulsivity (linked to the top-down system) were affected by fast-paced programs.

In the CPT task, a salience stimulus triggers an automatic orienting response, engaging the bottom-up attention [31, 51]. This system is similar to the processing of fast-paced program stimuli, leading to quicker responses. Conversely, tasks requiring attention allocation based on instructions involve goal-based processing (top-down system), demanding more effort and resulting in a slower response [39]. In the Attention Networks Task (ANT), the orienting network involves attention shifting in response to relevant stimuli. However, it is unable to evaluate the bottom-up and top-down attention systems separately [52]. The findings of this task indicate that 4-year-old participants watching a slow-paced program showed higher and quicker performance in the orienting network. However, results from 6-year-olds were opposing. This result aligns with reduced error rates in children exposed to a fast-paced program. Furthermore, no discernible distinctions emerged in the executive control network, indicative of top-down attentional processes.

While it is assumed that the mechanisms of the attention system and the allocation of resources can explain the observed results, not all findings can be accounted for through this framework. First, it was hypothesized that the engagement of the bottom-up attentional system following exposure to a fast-paced program would tax executive resources [13] and affect tasks that need the top-down processing system. However, Bushman and Miller's [30] research contradicted this notion, indicating that rapidly presented stimuli exclusively stimulate sensory processing rather than the prefrontal cortex. Consequently, the fast-paced program exposure does not involve prefrontal neurotransmitters. Thus, this program is unlikely to impact subsequent tasks reliant on the prefrontal cortex (top-down processing). In light of these, there is a need for further exploration of the proposed hypotheses concerning the mechanisms that underlie the impact of program pacing on attention.

#### Fantasy and pacing interaction

Kostyrka-Allchorne et al. [39] uncovered a positive impact resulting from the interaction between fantasy and pacing. This result implies that when watching a fast-paced TV program, improvements in bottom-up attention may be observed, but only if there are no features in the program that trigger executive processing (fantasy stimulus). This discovery underscores the significance of examining the interaction between these factors rather than analyzing them in isolation.

#### Fantasy

The exploration of fantasy's impact on attention has been limited to a single study conducted by Kostyrka-Allchorne et al. [39]. The assumption is that watching a fantastical program heightens orienting responses and triggers bottom-up processing, which continues in subsequent tasks [14]. Consequently, similar to the impact of the fast-paced program, a quicker response in bottom-up attention tasks can be seen. Alternatively, comprehending fantasy features might require extensive engagement in executive processes. Due to the limited capacity of these resources, they could become overwhelmed [14], leading to diminished performance in tasks related to top-down attention. However, the outcomes of the Continuous Performance Task (CPT) do not reveal any difference between the results of children in the high and low fantasy groups. This underscores the necessity for further research in this particular domain.

### Inhibitory control

#### Pacing

Exploring pacing's potential influence has been limited to just two studies conducted by Fan et al. [25] and Kostyrka-Allchorne et al. [39]. These studies failed to identify any significant effects of pacing on inhibitory control. The study results contradict the assumptions made about the underlying aspects. Yet, these findings align with Bushman and Miller's [30] study. Therefore, it can be inferred that the pacing feature, possibly because it does not engage the prefrontal cortex, does not impact subsequent tasks reliant on the top-down system, such as inhibitory control.

#### Fantasy

There was a more extensive body of research that examined the impact of fantasy. The collective of these studies from Fan et al. [27], Jiang et al. [26], Li et al. [42], and Rhodes et al. [10] have consistently revealed a trend: exposure to fantastical TV programs leads to a reduction in inhibitory control. However, Kostyrka-Allchorne et al. [39] diverged from this trend as the only one that did not conform. It's worth highlighting that Jiang et al. [26] indicated the potential for varying impacts of mild fantasy, suggesting a non-linear relationship between the level of fantasy and the EF component such as inhibitory control.

In these studies, a variety of tasks were employed to evaluate inhibitory control. Li et al. [42] used the go-no-go task to measure response inhibition. Jiang et al. [24] employed the flanker task, whereas Rhodes et al. [10], Fan et al. [27], and Kostyrka-Allchorne et al. [39] used the Day-Night task based on the Stroop paradigm to measure interference control. Although both response inhibition and interference control are considered aspects of inhibitory control, their measurement approaches exhibit differences [53]. Notably, the variation in tasks employed does not account for the differences in results, as evidenced by the Kostyrka-Allchorne et al. [39] study, which, despite using the Day-Night task like the other two studies, reported results contrary to the overall trend.

Additionally, the processing of fantastical events depicted in cartoons appears to trigger distinct neural circuits, particularly the anterior cingulate cortex (ACC), which is associated with inhibitory control [54, 55]. Through information processing theories, it seems that fantastical animations require increased cognitive resources in the ACC, resulting in a temporary depletion of resources available for subsequent tasks [14, 34]. However, Kostyrka-Allchorne et al. [39] suggested that this trigger leads to enhanced performance.

### Working memory and cognitive flexibility

#### Pacing

The investigation into the impact of pacing remains limited to a single study. In this study conducted by Fan et al. [27], it was established that pace does not exert a significant effect on working memory and cognitive flexibility. Similar to previous research, this result indicates that pacing does not affect tasks related to the top-down system.

#### Fantasy

Jiang et al. [26] did not identify any significant impact on working memory. However, both Fan et al. [27] and Rhodes et al. [10], in their respective studies, observed a decline in working memory after exposure to fantasy TV programs. Upon looking at the tasks used by these articles to measure working memory, we find that Jiang et al. [26] used List sorting working memory, while Rhodes et al. [10] and Fan et al. [27] used backward digit span. Regarding cognitive flexibility, Wang and Moriguchi [43] did not observe a fantasy effect on flexibility, whereas Fan et al. [27], Jiang et al. [26], and Rhodes et al. [10] identified a negative impact of fantasy. The task employed by Wang and Moriguchi [43] to measure flexibility was the same as that used by Jiang et al. [26] and Rhodes et al. [10], the standard Dimensional Change Card Sort Task. Only Fan et al. [27] utilized a different task, the Flexible Item Section. These two tasks are almost the same, and there is no discernible difference in their impact on the results. Although the fantasy cartoon used in Wang and Moriguchi's [43] study features only seven fantasy events, this quantity is significantly lower than the programs used in other studies and is closer to the number of programs considered realistic.

### Higher-order EFs

#### Pacing

Higher-order EFs have received limited attention within the context of TV content effects. Only Rose et al. [29] measured the influence of pacing on problem-solving, revealing no significant differences, aligning with similar findings from other studies.

#### Fantasy

Research on the impact of fantasy is also lacking. Rhodes et al. [10] explored how fantasy impacts planning and found no discernible effect. Notably, our review reveals a gap, with no additional studies examining the influence of fantasy on other higher-order EFs. This highlights the need for further investigation into the broader effects of fantasy on various aspects of EF.

### Broader dimensions of EF

#### Pacing

In addition to studies focusing on specific components of EF, there have been investigations into EF in a broader way. For Cool and Hot EF, Lillard and Peterson [13] reported a negative impact of pacing, while Lillard et al. [14] did not observe any. Moreover, in the realm of general EF, only Sanketh et al. [28] delved into the effect of pacing on EF (motor EF), revealing a negative influence.

#### Fantasy

Examining the impact of fantasy on Cool EF, two studies, Lillard et al. [14] and Li et al. [41], found a negative influence. However, in the context of Hot EF, Lillard et al. [14] did not identify any discernible impact.

Together, drawing conclusive findings about the effects of pacing on attention and fantasy on attention and components of EF is challenging due to conflicting results or a limited number of studies. As we consider studies with contradictory results, it becomes evident that various influential factors come into play. These factors encompass the content of the programs, individual child characteristics, environmental influences, and the methodologies employed in the studies. Despite some attempts to control for specific factors, it remains clear that the presence of these variables can contribute to discrepancies between study outcomes. Consequently, there is a pressing need for more comprehensive investigations that carefully consider and account for these variables. This approach would lead to a more nuanced understanding of the relationship between TV program features and children's attention and EF. Future research should address these gaps and consider a broader range of factors to arrive at more conclusive insights.

### Influential factors

#### TV program content

In studies focusing on the immediate effects of TV programs, the content emerges as a determinant of its impact. Hence, it becomes crucial to ensure that other content-related aspects, apart from the independent variable, are identical across experimental groups. However, when utilizing existing TV programs, maintaining control over this factor becomes exceedingly challenging. Distinct programs possess varying characteristics, with some designed for educational purposes for children, while others primarily serve entertainment. This dichotomy of educational versus entertainment is a trait that studies have identified as influential in their impact on EF (for more details, refer to Fan et al. [56]). Another salient feature of programs is the type of language employed within them. Language intricately links to EF, and the processing of unfamiliar vocabulary could potentially impose greater cognitive demands on children, especially evident in the context of fantastical TV programs [57].

Only a limited number of studies successfully matched the inherent content features of programs by making their videos. For instance, Cooper et al. [12], Kostyrka-Allchrone et al. [38], and Kostyrka-Allchrone et al. [39] created a live-action adaptation of a storybook. However, these videos differed from the typical programs children encounter daily and the pacing measuring method varied between live-action videos and animations (such as changes in camera angles). Consequently, these discrepancies between live actions and animations can contribute to disparate outcomes. It appears that children exhibit greater attention to animated content compared to live-action programs [58]. Additionally, the quantity of fantasy events in programs identified as fantasy is a noteworthy factor in the research. For instance, in Wang and Moriguchi's [43] study, the fantasy program featured only seven events, placing it closer to realistic programs with four events rather than high-fantasy ones, which typically have more than 16 events. Moreover, Jiang et al. [26] indicated the potential for varying impacts of mild fantasy, suggesting a non-linear relationship between the level of fantasy and the EF component. In this study, a TV program categorized as mid-fantasy contained 17 fantasy events, a number close to those considered high fantasy in other studies. Meanwhile, the cartoon characterized as high fantasy in Jiang et al. [26] study featured 31 fantasy events, a level rarely included in other research studies. These variations highlight the importance of considering the quantity and level of fantasy events when examining their impact on children's attention and EF.

#### Individual child characteristics

Recent study reviews have prompted inquiries into the differential susceptibility of children to the influence of TV programs. An essential consideration in this context is the child's age, as previous research indicates a developmental trajectory of cognitive functions about age [59]. As a result, an exploration of age's role in the interaction between TV programs and attention or EF becomes imperative. Although some studies like Fan et al.'s [27] have addressed the influence of age, younger age groups have yet to be incorporated into this line of investigation. Another dimension pertains to personality traits, which can modulate a child's responsiveness to their environment, including environmental sensitivity (SPS) [60, 61].

#### Environmental characteristics

The surrounding environment and its attributes constitute factors that can influence the impact of programs on attention and EF. One noteworthy environmental factor is SES, a determinant of the family's standing. In correlational studies, SES has emerged as a variable in moderating the relationship between TV program exposure and EF [62, 63]. Thus, an increased emphasis on assessing the role of SES within experimental designs is warranted.

#### Study methodologies

Beyond considerations encompassing TV content and child characteristics, the methodological approaches adopted in studies exert a noteworthy influence. Regarding this matter, some studies have omitted pre-test assessments due to the novelty of the EF measurement tools. Therefore, the analysis of post-TV program exposure changes becomes more intricate within these studies. On the other hand, attention and executive functions cover a wide range of aspects and can be measured using multiple instruments. The tools employed in existing literature serve distinct purposes and measure specific aspects of these cognitive functions. This heterogeneity in the selection of these tools can contribute to the contradictions observed in the study results. Therefore, future researchers must exercise greater caution in selecting their assessment instruments. Adopting a more consistent approach to measuring attention or different components of EFs may enable more efficient research.

Furthermore, studies examining the impact of pace employ various methods to measure the pacing of TV programs. For instance, some research utilizes the Sense Detector app [13, 14], which assesses the frames rather than the scenes of a program. Consequently, the numerical representation of a program's pace may differ when using this app compared to when the coder counts scenes [27] or employs tools to edit and accelerate the program [29]. This variability in measurement methods introduces the possibility that a program deemed fast-paced in one study might be categorized as having an average pace when using a different measurement approach. This underscores the importance of standardizing methods for assessing program pace to enhance consistency across studies and ensure accurate interpretations of the findings.

These multifaceted factors, collectively contribute to the intricate landscape shaping the relationship between TV program features and children's EF. Gaps within the existing body of research underscore the necessity for more comprehensive investigations that meticulously account for these variables.

## Limitations

Several limitations are noteworthy within the scope of this review. To initiate, it does not encompass unpublished studies or student theses that may have explored the pertinent question. This decision aligns with the established inclusion criteria to uphold a standard level of study quality. Additionally, during the process of identifying relevant studies, no search was conducted on gray literature platforms. Another limitation arises from the failure to report the scores of pace and fantasy assigned to the TV programs in the studies. These scores are crucial for categorizing programs as fast-paced or slow-paced and determining the level of fantasy. The absence of these numerical scores in some studies has made it difficult to quantify and compare the pacing and fantasy across the reviewed literature. Moreover, this review only looks after findings from experimental studies that investigated the short-term impact of TV programs on children, while this study design has limitations. Experimental studies have challenges in generalizing findings to real-world situations, and observed short-term effects may not transform into long-term ones [11, 27]. Although, these short-term changes from experimental studies can be significant intrinsically [13, 14]. For instance, recent studies have indicated an increase in the use of media by kindergarten and preschool teachers in the classroom [64, 65]. Using these contents, such as TV programs, can have afterward effects on classroom learning conditions [66].

## Conclusions

**Fig. 2:**
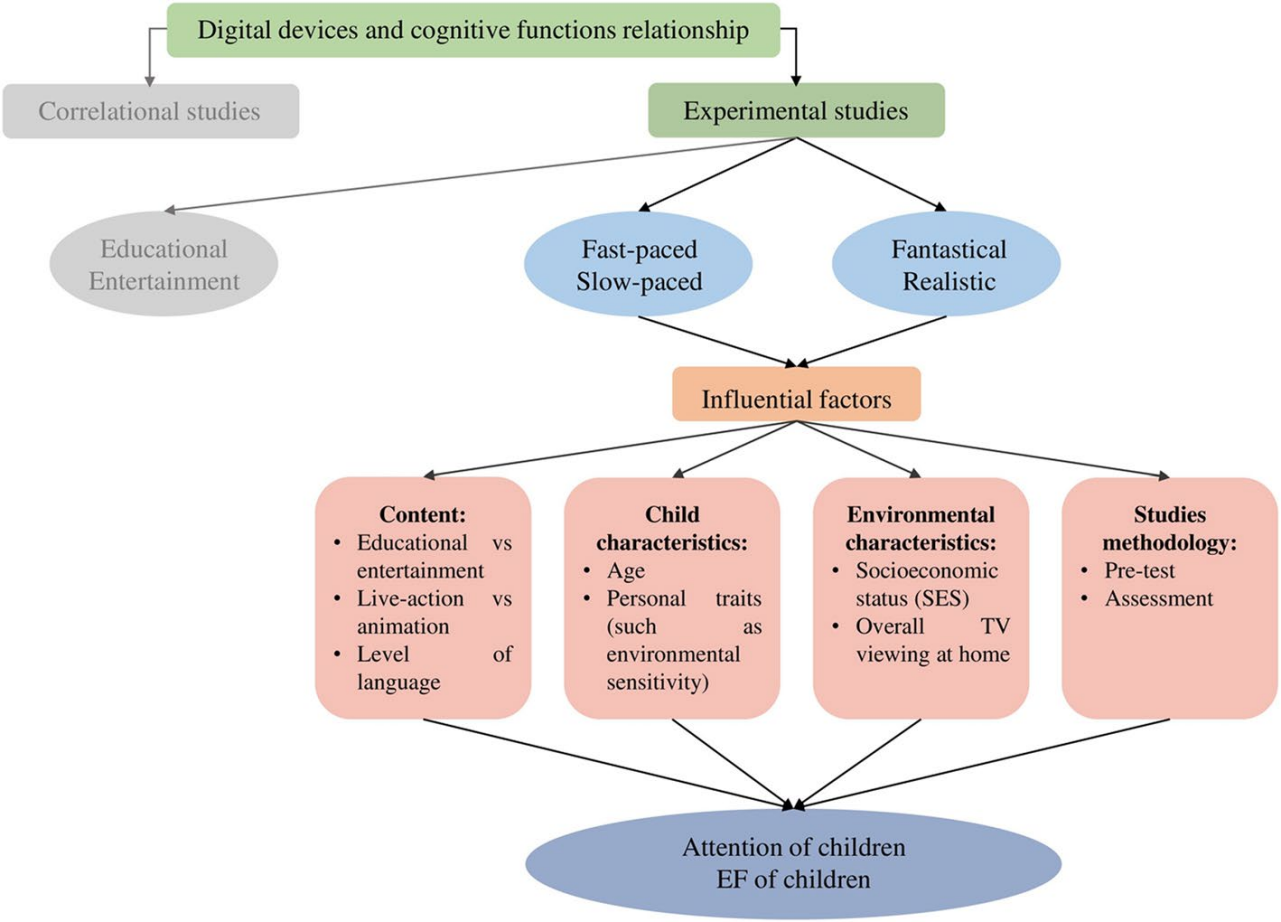
Conceptual map of the relationship between TV programs' pace, fantasy, and children's attention and EFs

In summary, this systematic review significantly advances our understanding of the intricate relationship between TV pace, fantasy, and their impact on children's attention and executive functions (EFs). For a visual representation of these relationships, please refer to Fig. 2. Concerning attention, there were limited studies available to conclude the impact of fantasy. Within the context of bottom-up attention, the influence of pace is discernible, although its mechanism remains elusive and exhibits variability across studies. On the contrary, there is no clear evidence of a pacing effect on the top-down system. Combining insights from experimental studies reveals the intricate ways TV programs influence specific aspects of EF. For instance, inhibitory control appears to be negatively impacted by the presence of fantastical events. Moreover, the complex interplay among factors such as content, child characteristics, environment, and methodology underscore the critical need for further comprehensive and nuanced investigations into this domain and its underlying mechanisms. As our understanding of this intricate relationship deepens, future research will play a pivotal role in guiding the development of informed guidelines for media consumption and its potential effects on children's cognitive development.

## Data Availability

All relevant data are within the paper.

## Abbreviations

EF: Executive function
SES: Socioeconomic status
CPT: Continuous Performance Task
ACC: Anterior cingulate cortex
